# Cultivating insight and engagement: exploring the role of Trait Emotional Intelligence in Chinese art education

**DOI:** 10.3389/fpsyg.2024.1372717

**Published:** 2024-06-19

**Authors:** Cong Wang

**Affiliations:** Jiangxi Police Institute, Nanchang, Jiangxi, China

**Keywords:** insight orientation, art education, Chinese students, Trait Emotional Intelligence, academic engagement

## Abstract

**Background:**

The present study focuses on the unique role of insight and Trait Emotional Intelligence in the realm of art education in China. Insight, traditionally associated with clinical outcomes such as mastering symptoms, developing adaptive behaviors, and enhancing quality of life, is newly contextualized in this study within the framework of art education. The expansion of insight research into areas like Social Psychology reflects its relevance beyond clinical settings, particularly in educational environments where creativity and emotional intelligence are pivotal.

**Aim:**

In Chinese art education, insight is crucial not just for personal growth but also for professional development, integrating the understanding of emotions, culture, and artistic expression. This study explores how Trait Emotional Intelligence fosters insight and engagement in art students, underscoring its transformative impact on their educational and professional journey in the art world.

**Methods:**

Using a sample of Chinese art education students at University level (*N* = 881), that answered a quantitative self-report questionnaire, statistical procedures are applied to test the relationships between Trait Emotional Intelligence, Insight orientation, and students’ academic engagement.

**Results:**

In the structural model, the path from Trait Emotional Intelligence to Insight Orientation was significant, indicating a positive relationship. Students’ Engagement was significantly predicted by Insight Orientation and Trait Emotional Intelligence.

**Implications:**

This finding corroborates theoretical assertions that individuals with higher emotional intelligence are more inclined to have enhanced insight. The findings of the present study extend beyond the field of Art education, allowing us to provide a broad spectrum of social implications for Higher Education institutions.

## Introduction

Art education witnesses a transformative shift driven by the integration of psychological constructs like Trait Emotional Intelligence and Insight Orientation. This research delves into the theoretical underpinnings of Trait Emotional Intelligence and its role in art education, expanding into the domain of Insight Orientation and its empirical applications within educational settings, specifically focusing on Chinese art students’ engagement.

Trait Emotional Intelligence emerges as a critical lens to comprehend the intricate relationship between emotional self-efficacy and artistic expression. Defined by Salovey and Mayer (1990) and refined by Petrides and Furnham (2000), Trait Emotional Intelligence encapsulates a multidimensional perspective on how emotions influence human behavior, particularly in creative domains. Its relevance to art education is underscored by Gardner’s (2015) theory of multiple intelligences, which posits that emotional faculties are integral to artistic endeavors. Empirical evidence supports the hypothesis that students with heightened Trait Emotional Intelligence exhibit superior ability in articulating complex emotional narratives through their art (Farrington et al., 2019). Furthermore, the pedagogical implications of Trait Emotional Intelligence suggest a paradigm shift towards methodologies that prioritize emotional understanding and expression, fostering an environment conducive to creative exploration (Valente and Lourenço, 2022).

Insight Orientation, an extension of the discourse on self-awareness and reflective thinking, offers a complementary lens for examining the educational process. This concept highlights the significance of an individual’s capacity for deep, intuitive understanding of their emotional and cognitive experiences (Martinsen et al., 2016). Within education, Insight Orientation has been identified as a pivotal element in fostering critical thinking and problem-solving abilities, with research indicating that students possessing high levels of Insight Orientation are better equipped to navigate complex information and challenges (Parker et al., 2004; Robinson and Aronica, 2016). The interplay between Trait Emotional Intelligence and Insight Orientation in educational outcomes, particularly within the context of art education, represents an emerging area of inquiry, with recent studies advocating for the integration of insight-oriented pedagogies to enhance student learning and personal development (Schön, 2017).

This research focuses on Chinese art students’ engagement, reflecting on the multifaceted influences shaping their educational experiences. Engagement, characterized by a student’s active participation and emotional investment in learning, is critically examined in the context of Chinese art education. The literature highlights several determinants of engagement, including cultural heritage, globalization, pedagogical strategies, and technological advancements (Zhao et al., 2020; Halimi et al., 2021; Ma, 2021). Hence, the research objectives of this investigation are twofold. First, to test the influences of Trait Emotional Intelligence on Chinese art students’ engagement. Second, to provide empirical evidence about the mediating role of Insight Orientation and its significant contribution to enhancing student engagement in the context of art education.

### Trait Emotional Intelligence and its role in art education

Trait Emotional intelligence, a construct rooted in the psychological understanding of emotions and their impact on behavior, has increasingly been recognized as a pivotal element in the realm of art education. Originating from Salovey and Mayer’s (1990) seminal work, Trait Emotional Intelligence encompasses a personality trait, also referred to as emotional self-efficacy, and assessed via self-report instruments (Petrides and Furnham, 2000). This multidimensional understanding of emotional processes has significant implications for the field of art education, a domain where emotional expression and comprehension are central.

In the context of art education, Emotional intelligence is not merely an adjunct feature but a core component of artistic development and pedagogy. Gardner’s (2015) theory of multiple intelligences, which includes interpersonal and intrapersonal intelligences (components of Emotional intelligence), underscores the relevance of emotional faculties in artistic pursuits. Students with high Emotional intelligence are often more adept at expressing complex emotional landscapes through their art, a claim supported by contemporary research in arts education (Farrington et al., 2019).

Furthermore, the role of Emotional intelligence in art education extends to the pedagogical approaches employed by educators. Teaching methodologies that integrate Emotional intelligence principles foster an environment where students are encouraged to explore and express their emotions, thereby enhancing their creative capacities. This approach aligns with recent pedagogical trends emphasizing the importance of socio-emotional learning in the classroom (Durlak et al., 2011). Instructors skilled in Emotional intelligence can create a learning atmosphere that values empathy, self-reflection, and emotional expression, crucial for nurturing artistic talent (Valente and Lourenço, 2022).

The implications of Emotional intelligence in art education are also evident in the development of critical thinking and problem-solving skills. As students engage with art, they learn to interpret and analyze emotional content, a process that enhances their ability to understand perspectives different from their own. This aspect of Emotional intelligence, often termed ‘emotional literacy,’ is essential in developing a nuanced appreciation of art, fostering tolerance and empathy (Alemdar and Anilan, 2020).

Integrating emotional intelligence (EI) into art education offers significant benefits for student well-being and mental health. The therapeutic nature of art, coupled with an emphasis on understanding and managing emotions, equips students with coping mechanisms for stress and anxiety (Hoffmann et al., 2021). Art educators, by incorporating EI principles, can contribute to the holistic development of students, addressing both emotional and artistic growth (Kim and Kim, 2018). Beyond bolstering artistic expression and fostering creativity, EI plays a crucial role in socio-emotional learning and student well-being. This integration is fundamental in shaping individuals who are not only adept in artistic skills but also emotionally aware and capable (Huerta et al., 2017).

Based on the above revised literature, the following hypothesis is proposed:

*Hypothesis 1*: Trait Emotional Intelligence positively predicts Chinese Students’ Engagement on Art education.

### Insight orientation from its definition to current applications in educational settings

Insight orientation, an empirically grounded concept within the field of psychology, has garnered significant attention for its applications in diverse contexts, including educational settings. Originating from the broader constructs of self-awareness and reflective thinking, insight orientation refers to an individual’s capacity to attain a deep, intuitive understanding of personal experiences, emotions, and thoughts. This self-reflective process plays a relevant role in personal development and decision-making, a notion supported by a great amount of psychological research (Martinsen et al., 2016).

In educational contexts, the application of insight orientation significantly influences learning processes and outcomes. Insight, recognized as a critical component in achieving critical thinking, is increasingly valued in the contemporary educational paradigm (Wagner, 2010). Empirical evidence suggests that students with higher levels of insight orientation demonstrate enhanced problem-solving skills and are more adept at processing complex information (Parker et al., 2004; Robinson and Aronica, 2016). Moreover, the scope of insight orientation extends beyond cognitive abilities to include emotional and social competencies. The development of emotional intelligence, intrinsically linked with insight, positively influences academic performance and interpersonal dynamics within educational environments (Salovey et al., 2002). However, despite various proposed mechanisms explaining the impact of Emotional Intelligence on educational outcomes, there is a notable absence of recent studies exploring the mediating role of Insight Orientation in the relationship between Emotional Intelligence and desired student outcomes. This research extends the application of Insight Orientation, traditionally examined in work-related contexts (Gori et al., 2022), to the educational domain. Supporting this perspective, recent studies indicate that students with greater insight into their emotional states are more adept at managing the challenges and stressors inherent in academic settings (Molero-Jurado et al., 2021; Sha et al., 2022).

Insight orientation is gaining traction in educational circles, recognized by instructors and psychologists for its value in developing well-rounded individuals. Reflective journaling, mindfulness practices, and collaborative learning are increasingly employed to cultivate student insightfulness (Schön, 2017). The impact of insight orientation extends beyond individual students, influencing educational policy and practice. Recent reform discussions advocate for integrating insight-oriented learning objectives into standard curricula, emphasizing a more comprehensive approach that balances cognitive, emotional, and social learning (Wagner, 2010; Robinson and Aronica, 2016).

Based on the above revised literature, the following hypothesis is proposed:

*Hypothesis 2*: Insight orientation mediates the relationship between Trait Emotional Intelligence and Students’ Engagement.

### Chinese art students’ engagement

Chinese art students’ engagement has received considerable interest (Halimi et al., 2021). Engagement, defined as the degree of attention, curiosity, interest, optimism, and passion students exhibit in their learning process (Fredricks et al., 2004), is particularly interesting in the field of art education, where creativity and personal investment are keys of academic success.

Recent studies have shown several factors influencing the engagement of Chinese art students. Cultural aspects, deeply ingrained in the Chinese education system, play a significant role. The Confucian heritage, emphasizing respect for authority and rote learning, contrasts with the more explorative and expressive nature of art education (Zhao et al., 2020). This dichotomy often challenges students to reconcile traditional educational values with the creative demands of their artistic pursuits.

Another critical factor is the influence of globalization and exposure to diverse artistic traditions. Chinese art students today have unprecedented access to global art forms, thanks to technological advancements and international collaboration in education (Zhao et al., 2022). This exposure not only broadens their artistic horizons but also presents challenges in integrating these influences within the context of their native artistic traditions.

The pedagogical approaches adopted by art educators in China also significantly impact student engagement. There has been a gradual shift from teacher-centered methods to more student-centered approaches, which encourage personal expression and individual creativity (Huang and Zhu, 2020). This shift aligns with contemporary educational theories emphasizing student agency and active learning, which are crucial in art education for fostering engagement and motivation (Deci and Ryan, 2013). Additionally, technological integration in art education has emerged as a notable factor. The use of digital tools and online platforms has transformed traditional art practices, offering new mediums and methods for artistic expression (Ma, 2021). This digital transformation, while offering new opportunities, also poses challenges in terms of skill adaptation and the potential impact on traditional art forms.

Chinese art students’ engagement hinges on a complex interplay of cultural heritage, educational approaches, global trends, and technology. Established cultural norms collide with contemporary art practices, creating a dynamic tension within evolving art education. Analyzing these diverse elements is essential to cultivate well-rounded artists who excel in both traditional and modern forms, adapting to shifting artistic landscapes. This not only enriches their learning but also contributes to the progressive development of art education in China and internationally.

**Figure 1:**
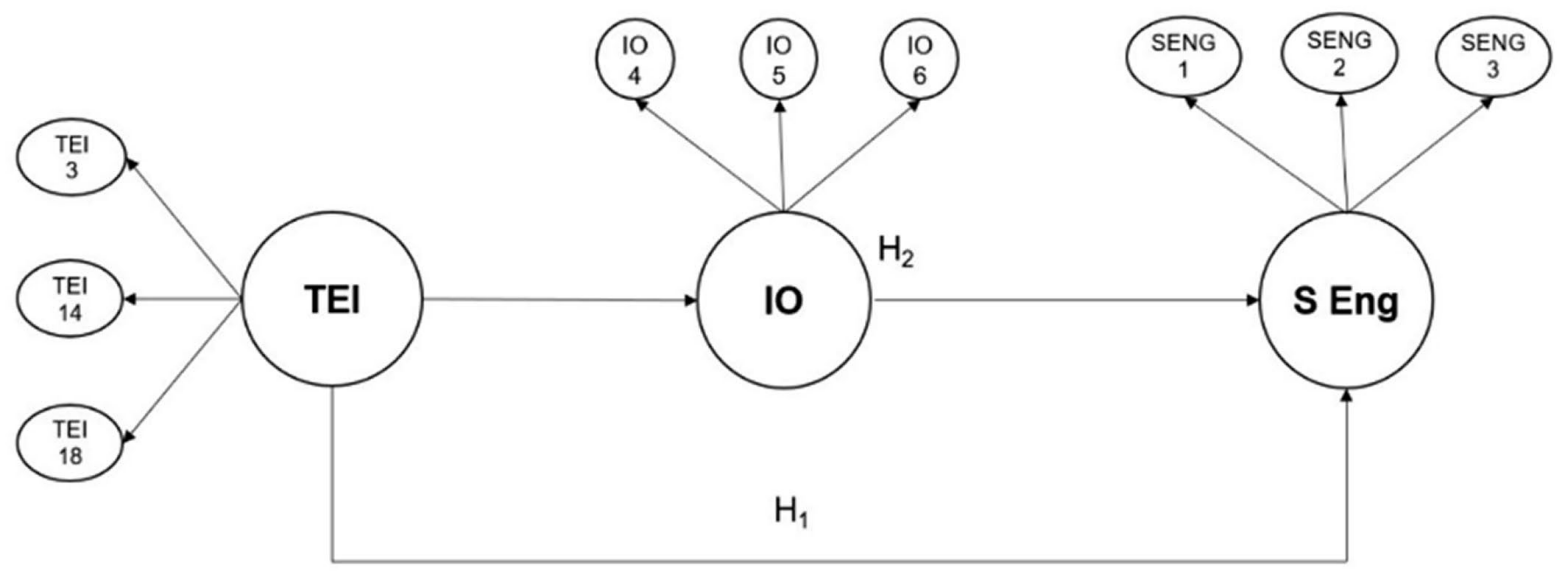
Research model and hypotheses for the study. TEI, Trait Emotional Intelligence; IO, insight orientation, S Eng, students’ engagement.

The full research model has been depicted in Figure 1.

## Methods

### Participants

The sample for the present study was recruited among students aged above 18 years enrolled in art study programs, as Bachelor degrees, Master degrees and Ph.D. in private and public institutions. Inclusion criteria were being an Art student in a Higher Education institution in China and being aged 18 years or more. Students taking art courses as complementary to their principal education program were excluded, as summer and winter schools, workshops, online courses or MOOCs (Massive Open Online Courses). Participants who entered the survey have been 986, but only 884 completed the questionnaire (response rate 89.6%). Three questionnaires have been eliminated due that failed to fill the 100% of the survey, giving a final sample of 881 participants. The gender distribution of the sample is a bit biased, 54.1% females, the majority of the participants were in a Bachelor’s Degree level (51.5%), while 40% were in Master’s studies and only 4.9% in Ph.D. programs. Regarding the distribution of the art specialties, 24.4% were enrolled in Calligraphy and traditional Chinese painting, 36.2% in Oil painting, Sculpture and Ceramics, 14.9% in Graphic and Fashion Design, 9.5% in animation and digital arts, and 7.7% in Photography and 7.2% in performance arts.

### Instruments

#### Trait emotional intelligence

The Trait Emotional Intelligence Questionnaire-Short Form (Petrides and Furnham, 2000) included 30 items, but the items 3, 14, and 18 do not contribute to any of the subtle factors, and only contribute to global EI, and can be used to calculate the trait EI score, as in previous studies (Feher et al., 2019). The English version of the items was: *In general, I am a highly motivated person, I find it difficult to adapt to change*, and *I find it difficult to get motivated by what I do.* In the present study, the Chinese version of the three items was used (Chiesi et al., 2020). Participants answered to items using a 7-point Likert scale ranging from 1(*Strongly disagree*) to 7 (*Strongly agree*).

#### Insight orientation

The present study utilized three items from the Insight Orientation Scale, a measure that assess participants’ insight capacity’ (Gori et al., 2015). The response format is a 5-point Likert scale from 1 (Not at all) to 5 (A great deal). The three items were selected to focus on the relevant dimensions for art students: Complexity, abstraction, depth (*I am aware of my inner thoughts about things*), level of consciousness (*I am in tune with my feelings*), and restructuring and behavioral change (*I can change my behavior when I realize that things are not going well*).

#### Students’ engagement

In the assessment of student engagement, the study utilized the abbreviated version of the Utrecht Work Engagement Scale (UWES-3), originally developed by Schaufeli et al. (2019), and available at the official website of UWES.^1^ This scale was modified for academic contexts. Comprising three items, the scale reflects key aspects of engagement: vigor, dedication, and absorption. The three items were as follows: *I feel energetic while creating art*, (vigor); *I am enthusiastic about developing my artistic skills*, (dedication), and *I am fully concentrated in the artistic process, even when there are distractions* (absorption). Participants answered to items using a 7-point Likert scale ranging from 1(*Strongly disagree*) to 5 (*Strongly agree*).

All the questionnaires have been provided to the participants in Chinese versions. When Chinese version was not available (Insight Orientation scale), the originally English survey was translated into Chinese. The translation was then double-checked through a back-translation process for accuracy. This process was carried out by the author, a native Chinese speaker who is also fluent in English. Additionally, the final Chinese translation has received approval from the author’s academic advisor. The complete scales in Chinese are included in Table 1.

**Table 1:** Chinese version of the items.

Trait emotional intelligence (Chinese version of 3 items)	Trait emotional intelligence (English version of 3 items)
总的来说，我是一个非常有动力的人	In general, I am a highly motivated person
我发现很难适应变化	I find it difficult to adapt to change
我发现很难被我所做的事情激励	I find it difficult to get motivated by what I do

Insight orientation (Chinese version of 3 items)	Insight orientation (English version of 3 items)
我意识到我对事物的内心想法	I am aware of my inner thoughts about things.
我与我的感受保持一致	I am in tune with my feelings.
当我意识到事情进展不顺时，我可以改变我的行为	I can change my behavior when I realize that things are not going well.

Students’ engagement (Chinese version of 3 items)	Students’ engagement (English version of 3 items)
在创作艺术时，我感到充满活力	I feel energetic while creating art.
我对发展我的艺术技能充满热情	I am enthusiastic about developing my artistic skills.
即使有干扰，我也会全神贯注于艺术创作过程中	I am fully concentrated in the artistic process, even when there are distractions.

#### Procedure

The present study was submitted to the Ethics Committee of the Jiangxi Police Institute for approval, previous to its implementation. Questionnaire was designed to be answered at the smartphones, and was implemented using Sojump as online platform for survey. The invitation to participate in the study was disseminated by social networks, as WeChat and Weibo, as well as Douban, due to its focus on culture, art and lifestyle, as Zhihu and Bilibili. The invitation included a description of the study objective, the voluntariness and anonymity of data collection, and the link to the survey at the end. When the participants entered the survey, they answered a prerequisite consisting in the informed consent question. If they agree, then they proceed to the survey. The total questionnaire was brief, and friendly to be answered in only one session, including only nine questions. The survey remains opened 3 weeks between March and April 2023.

#### Data analyses

Correlational analyses have been conducted using SPSS, while the Structural Equation Model analysis will be performed using JAMOVI with a sample size of 881 observations. The estimation method employed will be Maximum Likelihood. The model specification included three latent variables: Trait Emotional Intelligence (measured by items 3, 14 and 18), Insight Orientation (measured by items 4, 5, and 6), and Students’ Engagement (measured the three items of the UWES-3). The structural paths were defined as Insight orientation will be predicted by Trait EI and Students’ Engagement will be predicted by both Insight orientation and Trait EI.

The evaluation of model fit was conducted using several criteria. The Chi-square statistic and its corresponding *p*-value, the Adjusted Goodness of Fit Index (AGFI), the Comparative Fit Index (CFI), the Normed Fit Index (NFI), and the Incremental Fit Index (IFI). It has been posited that for these indices, values exceeding 0.90 denote a satisfactory model fit. Conversely, the Root Mean Square Error of Approximation (RMSEA) is deemed acceptable when its value is below 0.08, suggesting an especially strong fit between the model and the observed data. In order to specifically compare the effects of Trait Emotional Intelligence on Students’ Engagement, both total, direct and indirect, as mediated by Insight Orientation, further analysis with the Process 4.2. Macros for SPSS has been conducted, using the model 4 (Hayes, 2022). If zero is not included in the 95% bias-corrected confidence interval, the parameter is significantly different from zero at *p* < 0.05.

## Results

Table 1 shows the Pearson’s correlations between the variables included in the study. Thus, all correlations were significant (*p* < 0.001) excepts with Age, that only correlates with students’ engagement and in a negative way.

### Structural equation model (SEM)

The global assessment of the model displayed a Chi-square value of 186 with 24 degrees of freedom (*p* < 0.001), indicating a significant difference from the null model. The baseline model exhibited a Chi-square of 2064 with 36 degrees of freedom (*p* < 0.001). The fit of the model was assessed using several indices. The Standardized Root Mean Square Residual (SRMR) was 0.049 and the Root Mean Square Error of Approximation (RMSEA) was 0.087, with 95% confidence intervals ranging from 0.076 to 0.099, suggesting a moderate fit. The Comparative Fit Index (CFI) was 0.920, and the Tucker-Lewis Index (TLI) was 0.880, both indicating a good model fit. Other indices such as Bentler-Bonett Non-Normed Fit Index (NNFI), Relative Noncentrality Index (RNI), Bentler-Bonett Normed Fit Index (NFI), Bollen’s Relative Fit Index (RFI), Bollen’s Incremental Fit Index (IFI), and Parsimony Normed Fit Index (PNFI) also supported the adequacy of the model.

In the structural model, the path from Trait Emotional Intelligence to Insight Orientation was significant (*β* = 0.297, *z* = 5.99, *p* < 0.001), indicating a positive relationship. Students’ Engagement was significantly predicted by Insight Orientation (*β* = 0.316, *z* = 6.74, *p* < 0.001) and Trait Emotional Intelligence (*β* = 0.140, *z* = 3.21, *p* = 0.001). The *R*^2^ values for Insight Orientation and Students’ Engagement were 0.0881 and 0.1454, respectively, indicating the proportion of variance explained by the predictors in each case.

### Measurement model

In the measurement model, all observed variables significantly loaded on their respective latent constructs. For Trait Emotional Intelligence, the loadings were significant for all the observed items. The loadings for Insight Orientation were significant for all of the items, and, similarly, for Students’ Engagement.

Variances and covariance among observed variables and latent constructs were significant, indicating distinctiveness and relationships among the constructs. The intercepts for all observed variables were significant (*p* < 0.001), affirming the model’s intercept structure.

The reliability of the constructs was assessed using Cronbach’s alpha (α), omega hierarchical (ω₁), omega total (ω₂), and omega asymptotic (ω₃). The values for Trait Emotional Intelligence were *α* = 0.650, ω₁ = 0.668, ω₂ = 0.668, and ω₃ = 0.669, suggesting moderate reliability. For Insight Orientation, the reliability indices were *α* = 0.730, ω₁ = 0.750, ω₂ = 0.750, and ω₃ = 0.753, indicating good reliability. Students’ Engagement exhibited *α* = 0.717, ω₁ = 0.724, ω₂ = 0.724, and ω₃ = 0.711, also reflecting good reliability. The Average Variance Extracted (AVE) values for Trait Emotional Intelligence, Insight Orientation, and Students’ Engagement were 0.414, 0.510, and 0.482, respectively, further substantiating the adequacy of the measurement model (see Table 2)

**Table 2:** Pearson’s correlation matrix.

	Mean	S.D	Age	TEI	IO	SENG
Age	22.45	3.68	1			
Trait emotional intelligence	5.16	0.65	0.036	1		
Insight orientation	4.02	0.44	−0.008	0.434^**^	1	
Students’ engagement	4.08	0.65	−0.078^*^	0.306^**^	0.316^**^	1

*N* = 881; ^*^p < 0.05; ^**^p < 0.001. TEI, Trait Emotional Intelligence; IO, insight orientation, SENG, students engagement.

### Comparative fit indices

### Fit and comparative indices

The Goodness of Fit Index (GFI) was exceptionally high at 0.998, and the Adjusted Goodness of Fit Index (AGFI) was 0.994, indicating an excellent fit. The Parsimony Goodness of Fit Index (PGFI) was 0.443, reflecting a moderate level of parsimony. The McDonald Fit Index (MFI) was 0.912, further supporting the model’s fit. The Expected Cross-Validation Index (ECVI) was 0.279, indicative of the model’s potential generalizability.

**Figure 2:**
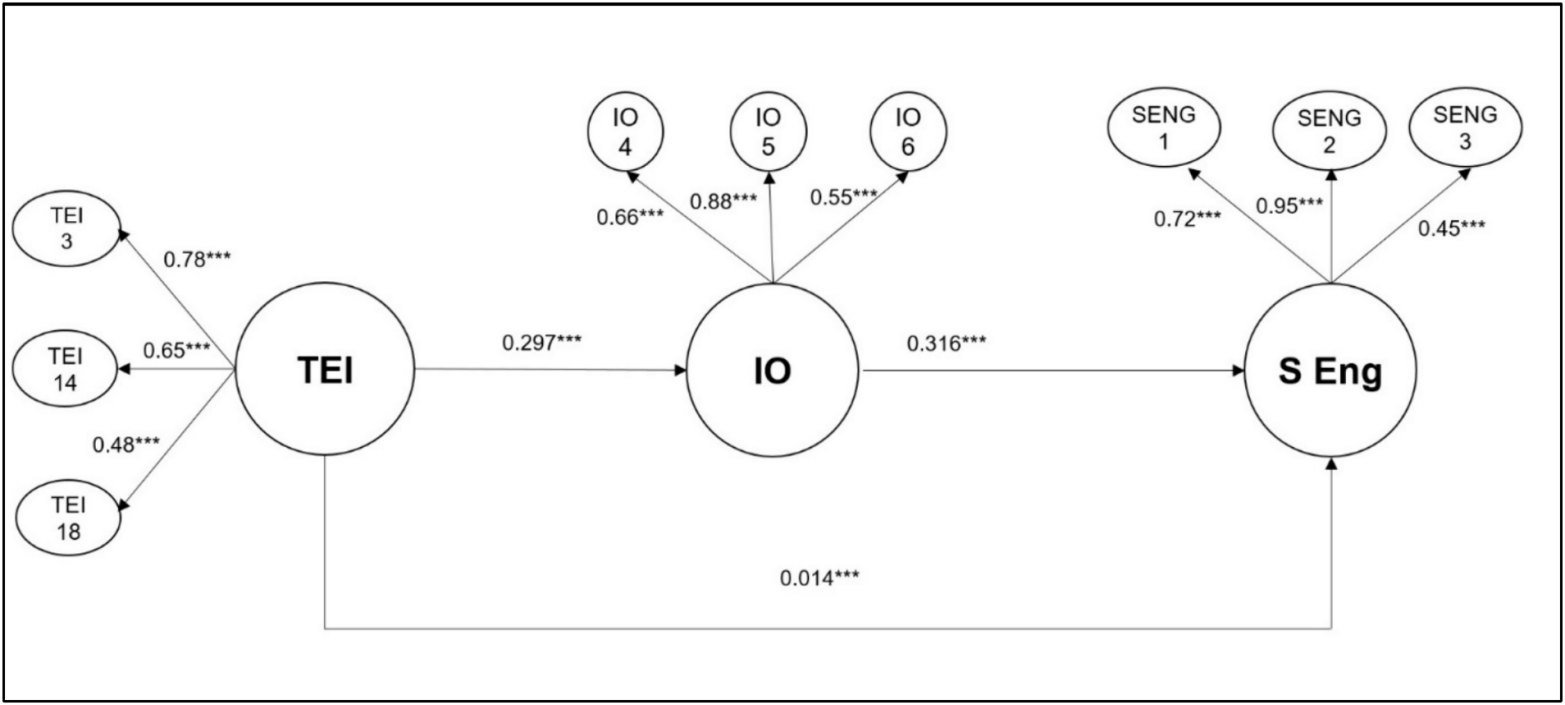
Standardized estimates for the measurement and the structural model. TEI, Trait Emotional Intelligence; IO, insight orientation, S Eng, students’ engagement.

The path model effectively captured the hypothesized relationships among the constructs. Path diagram (Figure 2) visually represents these relationships.

Hence, the SEM analysis demonstrated a well-fitting model with significant relationships among the constructs. The measurement model confirmed the validity of the latent constructs, and the structural model revealed meaningful paths among the variables. The model’s reliability and validity were supported by various indices, confirming its appropriateness for interpreting the relationships within the data.

Further analysis with the Model 4 allows to compare the total effect of Trait Emotional Intelligence on Students’ Engagement, with the direct effect and the indirect effect, mediated by Insight Orientation. As Table 3 shows, all the effects were significant. Moreover, the indirect effect, through Insight orientation were stronger than the direct effects, providing full support for the hypothesis.

**Table 3:** Total, direct, and indirect effects of Trait Emotional Intelligence (X) on students’ engagement (Y).

Total effect of Trait Emotional Intelligence (X) on students’ engagement (Y)					
Effect	SE	t	LLCI	ULCI	c Completely standardized
0.3070	0.0322	9.5446	0.2439	0.3701	0.3064

Direct effect of Trait Emotional Intelligence (X) on students’ engagement (Y)					
Effect	SE	*t*	LLCI	ULCI	c’ Completely standardized
0.2091	0.0349	5.9915	0.1406	0.2777	0.2088

Indirect effect(s) of Trait Emotional Intelligence (X) on students’ engagement (Y) mediated by insight orientation			
Effect	Boot SE	Boot LLCI	Boot ULCI
Insight Orientation 0.0979	0.0173	0.0646	0.1333

Completely standardized indirect effect(s) of Trait Emotional Intelligence (X) on students’ engagement (Y) mediated by insight orientation			
Effect	Boot SE	Boot LLCI	Boot ULCI
Insight Orientation 0.0977	0.0184	0.0635	0.1354

## Discussion

The purpose of this study was to examine the predictive role of Trait Emotional Intelligence on Students’ Engagement and the mediating role of Insight Orientation in this relationship within a cohort of 811 Chinese art students. The model’s findings underscore the significance of Emotional Intelligence in educational settings, suggesting that students with heightened emotional intelligence and a deeper understanding of their inner experiences are more likely to engage effectively in their learning environments. The structural model’s R-squared values for Insight Orientation and Students’ Engagement, indicative of the proportion of variance explained by the predictors, underscore the robustness of these relationships. This evidence highlights the substantial impact of Trait Emotional Intelligence and Insight Orientation on Students’ Engagement, emphasizing their importance in understanding student behavior in educational contexts.

The current results align with prior research highlighting the influential role of Trait Emotional Intelligence in both Western (Resnik and Dewaele, 2023) and Eastern cultures (Halimi et al., 2021; Alkhayr et al., 2022). Trait Emotional Intelligence has been linked to various processes, such as self-regulated learning strategies, tolerance for uncertainty, and learner autonomy, to elucidate its stable and significant impact on student success (Albani et al., 2023; Zadorozhny et al., 2024).

Furthermore, the SEM analysis elucidates a notable positive relationship between Trait Emotional Intelligence and Insight Orientation, corroborating the latter’s mediating role in the connection between Emotional Intelligence and Engagement. This finding corroborates theoretical assertions that individuals with higher emotional intelligence are more inclined to have enhanced insight. This insight is seemingly pivotal in augmenting Students’ Engagement, particularly in art education. In a related vein, some studies have recently explored the role of social and emotional Intelligence in specific forms of art education, as Music (Novska, 2022). Art education, a domain where critical assessment (Lukaka, 2023), complex thinking meta-competence (Silva Pacheco, 2020), and innovation are paramount, has seen recent studies where applications of Insight Orientation could be highly beneficial. Additionally, the challenges posed by developments in Artificial Intelligence design (Zhang et al., 2022), distance learning systems, and virtual reality technology (Jiawei and Mokmin, 2023) in art education (Zhang et al., 2023) necessitate a nuanced understanding of the processes influencing student engagement. Despite the importance of these factors, there remains a scarcity of empirical research exploring student perceptions and outcomes in art education, particularly regarding the roles of insight orientation and engagement in their learning processes.

### Limitations of the current study and suggestions for future research

The methodology of the present study presents certain limitations that must be acknowledged. Firstly, the recruitment of participants through social networks may have introduced a selection bias. This mode of recruitment potentially excludes art students who are less active or have no presence on these platforms. Expanding beyond social media platforms to include other forms of outreach might help in obtaining a more representative sample. Additionally, future studies could provide more solid evidence supporting our finding’s patterns, as the positive relationships between Trait Emotional Intelligence and Students’ engagement mediated by Insight Orientation. Another limitation arises from the brief and concise nature of the questionnaire. While advantageous for participant convenience and higher response rates, the limited number of items may have restricted the depth and breadth of the data collected. This brevity, especially in measuring complex constructs like Trait Emotional Intelligence and Insight Orientation, might not fully capture the nuanced aspects of these constructs. Future research could benefit from employing a more comprehensive set of items (Grant et al., 2002). This would be particularly beneficial for capturing the complexity of Trait Emotional Intelligence and Insight Orientation in the context of art education (Martinsen et al., 2016). Lastly, considering the unique context of art education, future studies might examine how these constructs play out in different scholar disciplines to understand better the specificity of these relationships across various education settings. This would significantly contribute to the literature on emotional intelligence and student engagement in the realm of higher education.

### Intervention programs with art educations students and suggestions for teachers

The following suggestions can be offered, grounded in the findings of the study. Art curricula can be enriched by integrating modules focused on developing emotional intelligence. These modules should be designed to enhance self-awareness, empathy, emotional regulation, and interpersonal skills (Jenney, 2012b). Practical activities like group discussions, role-plays, and reflective exercises can make these modules interactive and impactful (Huang and Zhu, 2020). In a related vein, teachers should be provided with professional development opportunities focused on emotional intelligence can equip them with the skills to better understand and support their students’ emotional needs. This could include training in identifying emotional cues in students, understanding the role of emotions in learning, and strategies for creating an emotionally supportive classroom environment (Jenney, 2012a). For instance, incorporating exercises that require students to express their emotions through art can bridge the gap between theoretical understanding and practical application. Similarly, conducting workshops that specifically target the development of insight orientation can be highly beneficial for art students (Omotoy, 2023). These workshops should focus on promoting self-reflection, cognitive complexity, and creative thinking. Activities could include guided meditation, analysis of artistic works from multiple perspectives, and exercises that challenge students to rethink and reinterpret their artistic creations (Zhou et al., 2023). Finally, mentorship programs where experienced artists or senior students mentor younger students can provide valuable support (Luo and Lau, 2020). These programs should focus not only on technical skills but also on the emotional and insightful aspects of art creation. Mentors can share their experiences, offer guidance on navigating emotional challenges in art, and provide feedback that fosters insight.

### Social implications for higher education institutions

The findings of the present study extend beyond the field of Art education, allowing us to provide a broad spectrum of social implications for Higher Education institutions.

Firstly, the study’s emphasis on insight in the context of art education underscores the value of deep self-awareness and the ability to critically reflect on one’s thoughts, emotions, and behaviors. For Higher Education institutions, this means creating an environment where students are not only academically proficient but also emotionally intelligent and culturally aware individuals who can contribute positively to society (Xu et al., 2019).

Furthermore, the concept of Teaching Emotional Intelligence expands the focus beyond individual development. It encourages institutions to create an environment where emotional intelligence is embedded throughout the curriculum. This approach enhances student engagement, creativity, and resilience, particularly relevant in Chinese art education where emotions, culture, and artistic expression are deeply intertwined. By embracing these dimensions, institutions can cultivate the next generation of artists who are not only technically skilled but also emotionally and culturally competent (Jin and Ye, 2022).

Moreover, fostering insight and Teaching Emotional Intelligence can significantly impact students’ professional development (Pong and Leung, 2023). Art education programs that incorporate these elements equip students with the emotional and cognitive skills needed to navigate the complexities of the art world and succeed in their careers (Wang et al., 2022). This not only benefits individual students but also contributes to the art industry itself, which thrives on innovation, emotional depth, and cultural understanding.

## Conclusion

This empirical article underlines a pivotal advancement in our comprehension of how Trait Emotional Intelligence, Insight Orientation, and Student Engagement intertwine, especially within art education. By methodically investigating these dynamics, the study not only addresses but also illuminates the research objectives and questions posited at its inception. It conclusively demonstrates that Trait Emotional Intelligence is paramount in influencing students’ engagement levels. This relationship is especially pronounced in art education, where emotional intelligence not only underpins students’ academic engagement but also enriches their creative endeavors and outcomes.

The findings highlight the instrumental role of emotional intelligence in cultivating an enhanced insight among art students. This enhanced insight, in turn, acts as a catalyst for increased academic engagement. It underscores that insight orientation goes beyond theoretical understanding, serving as a vital, actionable strategy for art students to augment both their creative processes and academic performance.

Moreover, the study transcends the academic spheres of psychology and education, offering tangible guidelines for crafting educational interventions aimed at nurturing emotional intelligence among art students. This global contribution is significant, as it provides educators and policymakers with evidence-based insights to support the development of curricula and teaching methods that emphasize emotional intelligence.

In essence, this study bridges a critical gap by linking emotional intelligence research with its practical implications in education, particularly in the art domain. This connection not only enriches our theoretical knowledge but also paves the way for practical applications, marking a significant stride towards enhancing student engagement and success in art education through the lens of emotional intelligence.

## Data availability statement

The raw data supporting the conclusions of this article will be made available by the authors, without undue reservation.

## Ethics statement

The studies involving humans were approved by Ethics Committee of the Jiangxi Police Institute. The studies were conducted in accordance with the local legislation and institutional requirements. The participants provided their written informed consent to participate in this study.

## Author contributions

CW: Conceptualization, Data curation, Formal analysis, Funding acquisition, Investigation, Methodology, Project administration, Resources, Software, Supervision, Validation, Visualization, Writing – original draft, Writing – review & editing.

## Footnotes

^1^ https://www.wilmarschaufeli.nl/publications/Schaufeli/Tests/UWES_CN_9.pdf
